# Outcomes of a four-year specialist-taught physical education program on physical activity: a cluster randomized controlled trial, the LOOK study

**DOI:** 10.1186/s12966-016-0388-4

**Published:** 2016-06-08

**Authors:** Rohan M. Telford, Lisa S. Olive, Thomas Cochrane, Rachel Davey, Richard D. Telford

**Affiliations:** Centre for Research and Action in Public Health, Health Research Institute, University of Canberra, Bruce, ACT 2617 Australia; Department of Psychology, Australian National University, Canberra, ACT 0200 Australia; Medical School, College of Medicine, Biology and Environment, Australian National University, Canberra, ACT 0200 Australia; Research Institute for Sport and Exercise, University of Canberra, Bruce, ACT 2617 Australia

**Keywords:** Physical activity, RCT, Physical education, Intervention, School

## Abstract

**Background:**

The objective of this study was to investigate the effect of a 4-year specialist-taught Physical Education (PE) program on physical activity (PA) among primary school children.

**Methods:**

A 4-year cluster randomised controlled trial was conducted in children (initially aged 8 years) from 29 primary schools (13 Intervention, 16 Control). Intervention students (*N* = 457) received 2 × 45 min PE lessons per week from specialist-trained PE teachers (68 lessons per year, 272 lessons over 4 years). Control group students (*N* = 396) received usual practice PE from generalist classroom teachers. PA during PE lessons was examined using the System for Observing Fitness Instruction Time (SOFIT). Pedometers (steps/day) were worn for 7 days each year, and accelerometers were worn concurrently in the final two years to assess moderate to vigorous (MVPA) and sedentary activity. Linear and generalized mixed models were used to determine differences in Intervention and Control student PA and the proportion of students meeting PA guidelines.

**Results:**

The intervention increased SOFIT-observed student MVPA during PE lessons by 6.5 mins (16.7 v 10.2, *p* < 0.001). Within intervention schools, participants increased their whole-day step counts (boys = 449 [CI,140 to 756]; girls = 424 [CI,222 to 626]) and minutes of MVPA (boys = 8.0 [CI,6.8 to 9.2]; girls = 3.5 [CI,1.7 to 5.4]) on PE days. However, compared to the Control group the Intervention did not: increase habitual steps/day or MVPA when averaged over 7 days; elicit greater improvements in these measures over time; or increase the odds of meeting step/day or MVPA recommendations. At age 11 years Intervention group boys were 20 mins less sedentary per day (380 [CI,369 to 391] vs 360 [CI,350 to 369]) and this effect was sustained at age 12 years.

**Conclusions:**

Well-designed specialist-taught PE can improve student PA during PE lessons. However for PE to be a significant contributor to improving habitual PA in pre-adolescent children, daily classes are likely to be required, and even this would need to be supplemented with a wider multicomponent strategy. Our finding of a reduction in sedentary time among Intervention boys warrants further investigation into the potential role PE could play in influencing sedentary behaviour.

**Electronic supplementary material:**

The online version of this article (doi:10.1186/s12966-016-0388-4) contains supplementary material, which is available to authorized users.

## Background

Physical education (PE) is considered to play an important role in the physical, social and psychological development of children during the primary school years. Of particular interest in response to reports that a large proportion of children worldwide are insufficiently active [1], is the health promoting role that PE might play through the provision of physical activity (PA).

A problem that has emerged in many primary schools is that PE is most commonly taught by generalist classroom teachers [2] who have little or no training in PE. Previous research has shown that these teachers face a number of barriers to teach PE including lack of confidence and motivation [3] and are unlikely to be sufficiently skilled to increase PA levels during PE lessons [4]. This may not only be affecting the frequency and quality of lessons [5], but also the way children experience and respond to physical activity opportunities.

The introduction of PE trained teachers into primary schools is one strategy that may improve overall PA. Although PA interventions in youth tend to yield only small benefits [6], improvements in PA during PE lessons are likely achievable; a systematic review of randomized controlled trials designed to increase PA during PE lessons found that students in PE intervention conditions spent 24 % more lesson time in moderate to vigorous activity (MVPA) compared to usual practice conditions [7]. While this is encouraging, concerns have been raised that intervention effects may be confined to the period in which the intervention is delivered (e.g. during the PE class) and overall effects on PA may not be sufficient to elicit health benefits such as improvements in body composition [6].

One factor, which is thought to increase the likelihood of providing positive health outcomes, is implementing long-term interventions [8]. For example, results from a 2-year enhanced PE curriculum found PE specialist and trained teachers can provide students with more PA during PE lessons than generalist classroom teachers [9]. Similarly, another randomized controlled trial which incorporated an intervention of two additional PE classes per week showed improvements in PA during school time over a 1 year period [10]. An important finding in this particular study was that benefits were not sustained when the intervention stopped, suggesting a longer-term or continuous intervention may be necessary to maintain health benefits [11]. These two studies aside, few long-term controlled trials using objective measures of PA have been conducted and the impact of PE interventions is yet to be clearly established [12].

The objective of this study was to investigate the effect of a 4-year specialist taught PE intervention on PA. The primary outcome measure of interest was student PA, but, importantly, the intervention was designed as a sustained educative program, part of the school curriculum, as distinct from shorter interventions designed specifically to increase PA or fitness. We hypothesized that: 1) students participating in the specialist-taught PE intervention would be more physically active during PE lessons than students receiving usual practice PE taught by generalist class-room teachers; 2) participation in the long-term intervention would increase overall (weekly) habitual PA and; 3) participation in the intervention would increase the number of students meeting PA recommendations of 12,000 steps [13] and 60 min of moderate to vigorous activity per day.

For the purposes of this study we refer to the specialist-taught PE program as the “Intervention” condition and usual practice PE delivered by generalist teachers as the “Control” condition.

## Methods

### Study design

This study is part of the multidisciplinary Lifestyle of our Kids (LOOK) project [14], a school-based cluster randomised controlled trial which commenced in 2005 in the Australian Capital Territory. The overall study incorporates measures of PA, fitness, motor control, psychological health, family influences, bone health, cardiovascular function, academic achievement and nutrition. The present investigation specifically examines PA measures collected over the four-year intervention period from age 8 (baseline) to age 12 years. The study was registered with the Australian New Zealand Clinical Trials Registry (ACTRN12615000066583).

### Recruitment and randomisation

Thirty government funded primary schools were invited to participate in the LOOK study by way of invitation to school Principals of which 29 accepted. Invited schools had similar school facilities and suburb level socioeconomic status (as estimated by the Australian Bureau of Statistics). All grade 2 children from the accepting schools were invited to participate by way of a parent information pack and consent form, from which 83 % accepted (*N* = 853). To be eligible to participate, parents were required to indicate that their child was willing to take part in PA and fitness activities. Approximately 90 % of the children had Caucasian parents, 8 % were of Asian descent, 1 % were Indigenous Australian or Polynesian, and no data on ethnicity were available for the remaining 1 %. Baseline measures were performed between September and December 2005 prior to randomisation. Thirteen schools (32 classes) were randomly assigned to the Intervention (13 being the number of schools the Intervention teachers could practically travel between and deliver 2 classes per week) and 16 schools (36 classes) to the Control group.

### Intervention

A summary of the Intervention program is shown in Additional file 1: Table S1. The Intervention was delivered by specialist PE teachers from the registered charitable organisation Bluearth Foundation (www.bluearth.org). Bluearth staff members were university trained and qualified PE teachers with further specialised training in the Bluearth approach. The Intervention consisted of 2 lessons of PE per week taught by a Bluearth specialist, which amounted to 90 min of the mandatory 150 min per week of PE required in the study jurisdiction. The remaining 60 min of required weekly PE was delivered at the discretion of the classroom teacher. The Intervention was delivered over four consecutive years between 2006 (Grade 3) and 2009 (Grade 6) during school time by 5 of the university trained PE teachers. Logbooks completed by the specialist teachers (data not shown), indicated that on average, 12 scheduled PE lessons per school were missed per year due to either public holidays or rescheduling by classroom teachers. Participants in the Intervention group therefore received on average 68 Intervention PE lessons per year, which amounted to 272 lessons over the duration of the four year study. The underlying philosophy of the Intervention program was to create an all-inclusive, enjoyable, challenging yet non-threatening environment for PA. The Intervention utilized the guided discovery method of teaching [15]. The objective of this approach was to encourage students to discover the answers to a range of physical movement problems and game strategies themselves, through experimentation and self-discovery. Specialist teacher logbook records indicated that lesson plans were, on average, made up mostly of game play (28 ± 12 mins), fitness activities (12 ± 10 mins), skill practice (8 ± 8mins) and core movements (5 ± 5 mins).

The Control condition schools continued with their usual PE program conducted by the generalist classroom teachers, none of whom were formally trained in PE. They reported via questionnaire that they adhered to the required 150 min of mandatory PE according to the curriculum requirements of the jurisdiction.

### Comparison of intervention and control school PE classes

A comparison of Intervention and Control PE classes was made using the system of observing fitness instruction time (SOFIT) [16]. This involved a group of trained observers recording the duration of PA of randomly selected children, teacher’s behaviour, and lesson context of PE lessons. Student PA during PE lessons was classified into minutes spent lying down, sitting, standing, walking and very active. SOFIT activities classified as walking and very active were combined to give an estimate of MVPA. Lesson context and teacher behaviour was categorised into time spent in management, general knowledge, physical fitness knowledge, fitness activity, skill practice and game play. In accordance with recommendations from a previous study [17], interval-by-interval intra-observer agreements in excess of 85 % were achieved on pre-recorded lessons prior to the collection of data in LOOK study schools. Six observers, five who were not otherwise involved in the study, completed all SOFIT observations over the four-year period. Observation times were arranged in advance with both Intervention and Control teachers.

To assess the number of PE lessons conducted per week, students were asked the following question: How many times per week do you usually do PE? (0 = never, 1 = once, 2 = twice, 3 = 3 times, 4 = 4 or more times per week). This question was part of a wider health questionnaire which referred to the current school year (grade 6, age 12 years). From these responses the average number of PE lessons conducted per week in Control and Interventions schools was calculated.

### Physical activity

PA was measured by pedometer to assess total daily physical activity (TPA), accelerometers to measure both daily moderate and vigorous activity (MVPA) and sedentary time (SED); and SOFIT as described above to estimate MVPA during PE lessons. In each year, seven consecutive days of pedometer (Walk 4 Life, Plainfield, IL, USA) data were recorded during September to December. Missing days of data were adjusted for as previously described [18]. In the final two years of measurement (age 11 and 12 years) accelerometers (Actigraph GT1M, Pensacola, FL, USA) were worn simultaneously, positioned on a belt around the waist adjacent to the pedometers. MVPA was defined as counts >2296 per minute and sedentary SED activity was defined as counts <100 per minute based on recommendations [19], using an epoch length of 60 s. The first day’s data were discarded to minimize any reactivity and days of accelerometer data were included if there were 10 or more hours of activity, an hour being considered invalid if there were more than 30 zero counts in a row. Data were analysed using Actilife version 6 (Actigraph, Pensacola, FL, USA). Accelerometers were not included at baseline due to budget and practical constraints, but were included in later years when the cost of the devices and participant numbers were lower.

### Pubertal assessment and socioeconomic status

Pubertal development was a self-assessment of “Tanner” stage (pubic hair, and genital development for males, breast development and date of menarche for females). In grade 4 this occurred at home with parental guidance, and in grade 6 the venue was a private room in a hospital setting with guidance from an experienced teacher or parent if they chose to attend. The socioeconomic status (SES) of each school suburb was accessed through Australian Bureau of Statistics [20]. We used a published SES index that designated advantage (high values) and disadvantage (low values) derived from variables such as income, educational attainment, and employment. The mean and standard deviation of this index for the suburbs in our study (1085 ± 40 and range 982–1160) was higher (with a smaller range) than Australia-wide (980 ± 84, 598–1251).

### Statistical analysis

All analyses were conducted in R [21]. The *lme4* [22] package was used to perform linear mixed effects analyses to examine group (Control or Intervention) differences in outcome measures (TPA, MVPA, SED). Our data were structured such that participants were nested within schools. As this clustered structure may result in non-independent data, whereby participants attending the same school may have a tendency to be similar in outcome variables, random effects terms for School, Grade and individual participant were examined. A significant likelihood ratio test comparing the null multilevel model with a null school-level model justified the inclusion of each of these variables as a random intercept term in all models. Because of known sex differences in PA, separate models were fitted for boys and girls and each model was adjusted for SES at the school level, and stage of maturation. To examine group differences in the number of students meeting PA recommendations generalized linear mixed effects models were performed to compare differences in the binary outcome variable (1, met PA recommendations and 0, did not meet recommendations). Routine model checking procedures, including visual inspection of residual plots were used to check for deviations from homoscedasticity or normality. To investigate differences in student activity, lesson context and teacher behaviour between Intervention and Control PE lessons, a series of Mann–Whitney *U* tests were conducted to account for the non-parametric nature of the data gathered through the SOFIT method.

## Results

### Participant characteristics

**Fig. 1:**
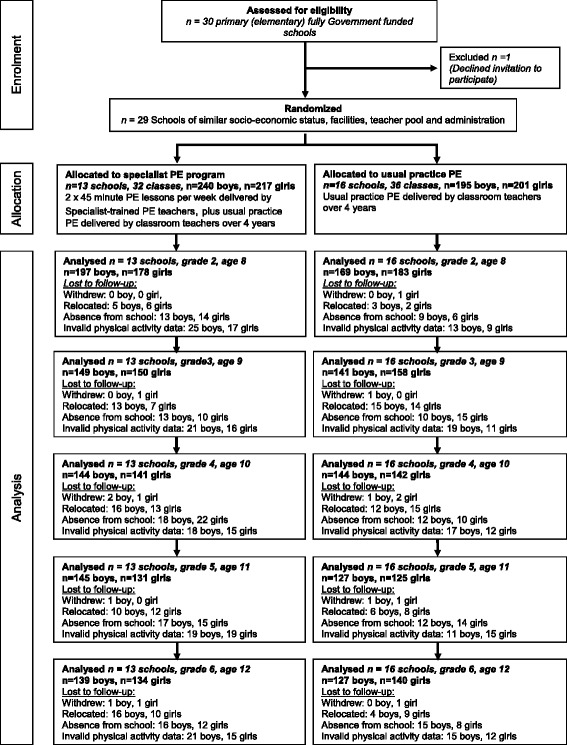
Study flow diagram of schools and participants through the 4 year intervention

Baseline characteristics are shown in Table 1. There were no significant group differences in height, weight, TPA or school SES measured at baseline between the Intervention and Control groups. As shown in Fig. 1, the number of observations varied with each test and the year of follow-up. Overall, 15 of the 853 children who gave consent to participate withdrew, 194 relocated to a school outside the study area and the remainder missing data was due to absence from school on test day or invalid physical activity data. Children who missed one or more assessments remained in the study and were included in the analysis, with the statistical model allowing for the incorporation of incomplete longitudinal data. There were no significant differences in the height, weight, TPA or SES for those children who remained in the study compared to those who did not complete the 4-year follow-up.

**Table 1:** Baseline characteristics (mean ± standard deviation) of the boys and girls in the specialist PE intervention group and usual practice PE control group

	Boys		Girls	
Characteristics	Intervention	Control	Intervention	Control
n	224	186	208	189
Age (years)	8.1 ± 0.4	8.2 ± 0.3	8.1 ± 0.3	8.1 ± 0.4
Height (cm)	130.5 ± 5.4	129.9 ± 5.6	128.4 ± 5.4	129.1 ± 5.3
Weight (kg)	28.9 ± 5.1	28.9 ± 5.4	28.5 ± 5.8	28.8 ± 5.7
Total Physical Activity, (average steps/day)	11,793 ± 3975	11,535 ± 3460	9273 ± 3328	9663 ± 3319
% meeting step per day recommendation	46	48	31	30

### Description of intervention and control PE classes

Over the four year study, 96 Intervention and 97 Control group PE lessons were observed using SOFIT. Results from these observations comparing group differences in physical activity are shown in Table 2. Results comparing lesson context and teacher behaviour are shown in Additional file 2: Table S2. Intervention PE classes were longer in duration than Control classes (47.1 vs 35.6 min, *p* < 0.05) during which time Intervention students performed more MVPA (16.7 v 10.2 mins, *p* < 0.01). Vigorous physical activity (VPA) was also higher among the Intervention group (6.4 vs 5.6 mins, *p* < 0.01). However, when expressed as a proportion, there was no difference in the percentage of lesson time spent in MVPA in the Control and Intervention groups (33.6 vs 35.4 %, *p* = 0.09) and Control students spent a greater proportion of lesson time in VPA (21.5 vs 14.6 %, *p* = 0.04). In accordance with longer lessons, Intervention students also spent more time sitting (7.9 v 3.3 mins, *p* = 0.006) and standing (18.9 v 11.3 mins, *p* < 0.01). With regard to lesson context, Intervention lessons were observed to contain more time spent in fitness activities (7.5 v 0.7 mins, *p* < 0.001), game play (11.2 v 3.1 mins, *p* < 0.01) and situations where students received general knowledge (8.4 v CP 1.0 mins, *p* < 0.01). In terms of teacher behaviour, the specialist trained Intervention teachers, compared to the Control group classroom teachers, were more actively engaged in PE lessons, spending more time demonstrating fitness (11.7 v 2.0 mins, *p* < 0.01, giving general instructions (17.0 v 10.3 mins, *p* < 0.01) and managing the class (8.8 v 5.9 mins, *p* < 0.05).

**Table 2:** Comparison of control and Intervention group physical activity during PE lessons, showing median (Mdn) and interquartile range (IQR) for the proportion of lesson time and number of minutes students in varying activity classifications

SOFIT activity category	Control PE lessons (*N* = 97)		Intervention PE lessons (*N* = 96)		
	Mdn	IQR	Mdn	IQR	*p*
Lesson Time					
Minutes^a^	35.6	9.7	47.1	10.6	0001
Sitting					
Minutes per lesson	3.3	(0.3,9.0)	7.9	(4.4,12.0)	0.006
Percentage of time per lesson	12.3	(2.7,44.1))	19.7	(11.5,29.3)	0.049
Standing					
Minutes per lesson	11.3	(7.8,16.2)	18.9	(12.7,24.2)	0.002
Percentage of time per lesson	44.4	(32.1,56.7)	43.6	(31.7,52.2)	0.448
Walking					
Minutes per lesson	4.3	(2.4,6.6)	9.2	(5.7,12.5)	0.004
Percentage of time per lesson	15.5	(8.5,22.2)	20.6	(14.3,25.1)	0.077
Very Active					
Minutes per lesson	5.6	(2.7,6.6)	6.4	(4.7,9.1)	0.006
Percentage of time per lesson	21.5	(10.2,33.9)	14.6	(10.7,20.2)	0.04
MVPA (Walking plus Very Active)					
Minutes per lesson	10.2	(7.8,12.8)	16.9	(11.6,20.2)	0.004
Percentage of time per lesson	33.6	(27.8,42.2)	35.4	(28.1,43.2)	0.09

^a^Lesson time presented as average minutes per lesson and standard deviation (SD). No data was recorded for the SOFIT “lying” category and is not shown. *PE* physical education, *SOFIT* system for observing fitness time, *MVPA* moderate to vigorous physical activity. NB Percentages of lesson time were calculated using median values and may not total 100 %

Results from the student questionnaire completed at age 12 years indicated that students in Intervention schools took part in more PE lessons on average per week compared to those in Control schools (M = 2.50 ± SD = 0.10 vs M = 2.01 ± SD = 1.04, *p* < 0.001).

### Intervention effects on physical activity

Results of the linear mixed regression analyses examining differences in TPA and MVPA between Control and Intervention groups, adjusted for school SES, stage of maturation and wear time (MVPA and sedentary time only) are shown in Table 3. No overall differences in average daily TPA, or change in TPA over time were found between students of either sex in Intervention and Control conditions. Examination of accelerometer data introduced in the final two years of the study indicated a trend toward a group difference in average daily MVPA for boys (DF = 1, F = 2.3, *p* = 0.130). This trend was characterised by higher MVPA among Intervention boys at age 11 years (53.3 vs 44.9 mins/day). However, this difference was not sustained at age 12 years. For girls the reverse was true. There was a weak trend toward a group difference in MVPA (DF = 1, F =1.59, *p* = 0.207) whereby MVPA was lower among Intervention girls at age 11 years (36.0 vs 41.6 mins/day) which was not sustained at age 12 years.

**Table 3:** Summary of linear mixed effects models comparing overall group (Intervention vs Control) and group-by-time differences in physical activity (adjusted means and 95 % CI)

	Age					Group	Group by Time
	Age 8	Age 9	Age 10	Age 11	Age 12	*P*	*P*
Total Physical Activity (steps/day)							
Boys Control	12134 (11469,12799)	11085 (10408,11762)	11354 (10675,12033)	11540 (10769,12311)	10238 (9370,11106)	0.763	0.131
Boys Intervention	12595 (11956,13235)	10456 (9796,11117)	11336 (10656,12015)	11276 (10578,11974)	10374 (9662,11087)		
Girls Control	10176 (9530,10824)	8844 (8214,9475)	9323 (8705,9941)	10322 (9563,11082)	9071 (8373,9770)	0.209	0.400
Girls Intervention	9733 (9100,10366)	8268 (7640,8896)	9256 (8685,9827)	9959 (9202,10716)	9141 (8424,9859)		
Moderate to Vigorous Activity (mins/day)							
Boys Control	NA	NA	NA	44.9 (40.2,49.7)	48.7 (44.1,53.3)	0.130	0.006
Boys Intervention	NA	NA	NA	53.3 (49.1,57.4)	49.9 (45.8,53.8)		
Girls Control	NA	NA	NA	41.6 (37.4,45.9)	35.6 (31.8,39.5)	0.207	0.076
Girls Intervention	NA	NA	NA	36.0 (31.8,40.1)	34.0 (30.1,37.9)		
Sedentary time (mins/day)							
Boys Control	NA	NA	NA	380.0 (369,391)	402.8 (391,414)	<0.001	0.761
Boys Intervention	NA	NA	NA	359.6 (350,369)	382.8 (373,393)		
Girls Control	NA	NA	NA	372.6 (361,384)	403.3 (392,414)	0.530	0.772
Girls Intervention	NA	NA	NA	372.5 (361,384)	401.3 (391,412)		

NA = Not available in early years due to unavailability of accelerometers at this time *CI* confidence interval

The majority of boys and girls in this study did not meet daily recommended levels of PA. Overall 43 % of boys achieved the recommended level of 12,000 steps per day and 31 % achieved 60 min of MVPA per day. The percentage of girls meeting PA recommendations was lower than boys. Only 26 % of girls achieved 12,000 steps per day and 16 % achieved 60 mins of MVPA per day. We found no evidence that the provision of two weekly PE lessons increased the odds of meeting either steps or MVPA recommendations when PA was averaged over 7 days. Results from the generalized linear mixed effects models found no difference in the odds of Intervention and Control boys meeting step (OR = 1.18, CI 0.87–1.61, *p* = 0.4) or MVPA (OR = 1.08, CI 0.8–1.44, *p* = 0.5) recommendations. Similarly no difference in the odds between Intervention and Control girls were found for step (OR = 0.82, CI 0.61–1.08, *p* = 0.16) or MVPA (OR = 0.80, CI 0.54–1.2, *p* = 0.4) recommendations.

### Intervention effects on sedentary time

In the final two years of the study significant group differences in average daily time spent sedentary were found for boys only. As shown in Table 3, at age 11 years Intervention boys spent 20 min on average less in sedentary classified activity and this difference was maintained at age 12 years. Non-significant group and group by time interactions indicated no difference in the amount of time spent sedentary between Control and Intervention girls, and no effect of the specialist PE program, respectively.

### Physical activity and sedentary time on intervention PE lesson days

As shown in Table 4, within Intervention schools, both boys and girls performed more TPA and MVPA on days when PE lessons were conducted in comparison with other school days. Boys took on average 449 more steps per day and engaged in 8 mins more MVPA on PE lesson days. For girls, an additional 424 steps per day and 3.5 mins MVPA were performed on PE lesson days compared to other schools days. Boys were 0.28 and 0.38 times more likely to meet step and MVPA per day recommendations respectively on PE lesson days. Girls were not more likely to meet either steps or MVPA recommendations than on normal school days. Both boys and girls were less sedentary on PE lesson days compared with other days of the week; boys spending 25 mins and girls 23 mins less time in sedentary classified activity on Intervention PE lesson days.

**Table 4:** Within Intervention schools analyses, comparing physical activity levels (adjusted mean and 95 % CI) on Intervention PE lesson days and usual practice school days, boys and girls analysed separately

	Intervention PE day	Usual practice day	Estimate	SE	P
Girls					
Total physical activity (Steps/day)	9862 (9354,10369)	9437 (8968,9907)	−424	163.4	0.009
% meeting step/day recommendations^a^	30	25	0.17	−0.05,0.39	0.139
MVPA (mins/day)	39.8 (35.7,43.8)	36.2 (32.5,39.9)	−3.53	1.40	0.013
% meeting MVPA recommendations^a^	15	14	−0.01	−0.42,0.41	0.97
Sedentary time (mins/day)	376 (363,389)	399 (387,411)	23.17	4.50	<0.001
Boys					
Total physical activity (Steps/day)	12261 (11720,12800)	11812 (11343,12281)	−448	206	0.02
% meeting step/day recommendations^a^	55	48	0.28	0.07,0.49	0.007
MVPA (mins/day)	62.2 (57.3,67.1)	54.2 (50.0,58.4)	−8.0	1.9	<0.001
% meeting MVPA recommendations^a^	35	31	0.38	0.07,0.69	0.02
Sedentary time (mins/day)	343.3 (331,356)	368.5 (358,379)	25.1	4.9	<0.001

^a^Statistics shown for physical activity recommendations are Odds Ratio, confidence intervals (CI) and probability-value (p); *SE* standard error, *MVPA* moderate to vigorous activity

## Discussion

This four-year randomized controlled trial examined the impact of a specialist-taught primary school PE intervention on objectively measured PA. We compared this Intervention, consisting of two 45 min classes per school week in grades 3 to 6, with current practice PE conducted by classroom teachers. The education-focussed PE Intervention was effective in increasing PA within the lessons themselves and Intervention participants were more active on PE days than on other days in the week. However this did not extend to eliciting a higher level of habitual PA (either MVPA or TPA averaged over 7 days); nor was it instrumental in increasing the number of children meeting recommended levels of PA. Of additional interest was that in the final two years of the investigation, there was some evidence of an intervention effect to decrease sedentary time in the boys.

PA outcomes of the Intervention were examined at three epochs: 1) during PE lessons, 2) days when Intervention PE was taught and 3) average daily PA. Firstly, with respect to PE lesson observations, the Intervention provided students with an additional 6.5 min of MVPA per lesson compared to Control school lessons. Lesson length seemed to be a particularly important factor in the delivery of the Intervention. SOFIT observations indicated that Intervention lessons were, on average, 11.5 min longer than Control lessons which allowed more time for students to perform MVPA. In light of the finding that Control and Intervention teachers dedicated a similar *proportion* of lesson time to MVPA, the conclusion could be drawn that generalist classroom teachers could improve student PA levels simply by conducting longer lessons. While this is possible, engaging students for longer periods of time may itself present a challenge to teachers untrained in PE, and may demand greater teaching competency and confidence. Indeed, previous research has shown that classroom teachers tend to conduct shorter lessons than trained PE teachers [9].

Several other differences in Control and Intervention teaching style were also observed in the present study. Intervention teachers were more actively engaged in lessons and allocated more time to game and fitness activities - each of these lesson elements were likely contributors to higher MVPA. Our findings are consistent with a 2-year randomized controlled trial in 4^th^ grade children [9] in which students in specialist-led PE conditions were observed using the SOFIT method to perform 14mins MVPA per PE lesson compared to 10 min in usual practice PE lessons. Similar to our findings, the specialist PE teachers conducted longer PE lessons than control group classroom teachers. When we consider these findings and those from the present investigation, the case that teachers with training in PE can deliver more physically active lessons compared to generalist classroom teachers is strengthened.

Secondly, with regard to PA on days PE was taught, we found that within Intervention schools, objectively measured student TPA and MVPA were higher on days when Intervention lessons were conducted. An increase of 8.0 mins MVPA for boys and 3.5 mins MVPA for girls on Intervention lesson days suggests that the PE Intervention had a small influence on PA behaviour. This finding is of smaller magnitude to those of a recent study of 9 year-old children in which accelerometer measured MVPA was 16mins higher on school days when PE was conducted [23] but nevertheless indicates that PE sessions can play a role in contributing to whole day activity levels.

Thirdly, despite evidence to suggest that the Intervention increased student PA during PE classes, there was no Intervention effect on daily TPA averaged over the entire week. Previous research has shown that increased PA attributed to PE intervention may not be sufficient to influence daily habitual activity. For example, a 1-year PE intervention in which students received two extra PE lessons compared to the control arm, increased accelerometer-measured TPA during school time, yet no differences in daily TPA when averaged over the 7 day period [10]. It is likely that in both this study and in ours, influences common to both Intervention and Control conditions, such as extracurricular sports participation were stronger drivers of habitual PA than the PE Intervention. Certainly in relation to the current cohort, sports club participation was high and a strong predictor of PA, as previously reported [24].

From the accelerometer data, it was apparent that habitual MVPA averaged over the week was higher among Intervention boys at age 11, but not 12 years. In the absence of baseline accelerometer data we cannot determine why this Intervention effect was observed, or whether these group differences in MVPA existed prior to age 11 years. Nevertheless, this finding is interesting because the reverse tended to occur in the girls at age 11 years, leading us to speculate that elements of the Intervention which increased MVPA for Intervention boys were ineffective in the girls. In any case, our findings, taken in conjunction with well-established findings that boys are more physically active than girls overall [1, 18], and during PE lessons [25], add weight to the premise that gender differences be carefully considered when designing PA interventions.

The incidence of insufficient PA in the current cohort is concerning. Overall only 43 % of boys and 26 % of girls met recommended levels of 12,000 steps per day [13] and only 31 % of boys and 16 % of girls achieved greater than 60 mins of MVPA per day. Contrary to expectations, provision of two teacher trained Intervention PE classes did not improve the odds of either boys or girls achieving step per day or daily MVPA recommendations. Instead, the small positive effects of the Intervention on PA appeared to be confined to days in which lessons were conducted. Even in the Intervention schools where boys and girls were more likely to meet daily step recommendations compared to other Intervention school days, it remains that only 55 % of boys and 30 % of girls met step per day guidelines on PE days. The proportion of children meeting MVPA guidelines on PE days was even lower, with only 35 % of boys and 15 % of girls meeting the recommended level of 60mins per day. This is a particularly concerning finding for girls, and, if PE is expected to play a strong role in helping children meet minimal PA requirements, our data highlight the need for PE lessons to be supplemented with additional opportunities for PA on a daily basis. Parents and teachers, at least in the current study jurisdiction, should not expect PE lessons, even if they were taught on a daily basis by trained PE teachers, to provide sufficient amounts of daily PA.

An interesting finding in this study was the effect of the Intervention to decrease sedentariness in the boys in the final two years of the study. Sedentary time was not only lower on PE lesson days in Intervention schools but also when averaged over the week (Intervention boys spent 20 mins less time sedentary on a daily basis compared to Control boys) suggesting the influence on sedentary time extended beyond PE lesson days. This was somewhat unexpected for two reasons: 1) the program did not set out to specifically target sedentary behaviour which is thought to be difficult to change due to strong habitual influences [26]; and 2) SOFIT classified sedentary activities (sitting and standing) were higher in Intervention PE lessons. This however was likely a reflection not only of longer PE lessons, but also of the educational nature of the Intervention where students were guided to reflect on their movements and perform stretching and balance exercises that require minimal movement. There has been growing interest in interventions that specifically target sedentary behaviour separately from those increasing PA, which has been motivated by reports stating that sedentary behaviour is associated with obesity [27] and metabolic risk in children [28]. Previous intervention strategies of this nature in young people have tended to focus on the involvement of family, behavioural interventions and electronic TV monitoring devices [26], so it is interesting that reductions in habitual sedentary time were associated with participation in a PE intervention in our study. While our data do not allow determination of which specific aspects of the intervention may have influenced sedentary behaviour, they imply that PE may be a useful vehicle by which to target reductions in sedentariness as well as increasing PA. Although our findings should be interpreted with caution due to lack of baseline accelerometer data, further investigations of this effect along with identification of mechanisms may be warranted.

In order to add context to our findings on PA it is important to emphasize that the specialist conducted PE program was designed as an educative program, part of the school curriculum, to be distinguished from interventions designed specifically to increase PA or fitness. While increasing PA was one objective, in contrast with physical training, the specialist taught PE was based on a broad set of educational principles, focussing on enjoyment, inclusivity, and the development of motor skills and social skills to encourage an ongoing physically active lifestyle. Bearing this in mind, this broad-based and sustainable program of PE has previously been shown to elicit improvements in academic achievement [29], reductions in insulin resistance [30] and attenuation of blood lipids [31], demonstrating that investigation of the benefits of PE should extend well beyond effects on habitual PA.

The present study has a number of strengths. The 4 year longitudinal, cluster randomised controlled design; the use of a statistical model to account for potentially confounding variables; and the incorporation of both week-long objective and in-class directly observed measures of PA. Another strong aspect was that we were able to maintain strong relationships with the schools, which enabled continued support of the research. In addition to these strengths, there were several limitations. The amount of PA in the Intervention classes may have been underestimated. The intervention involved activities that may not have been accurately detected by pedometers or accelerometers; activities borrowed from yoga, involving balance and isometric muscular contraction. These types of activities were often performed while sitting or stationary and may have been classified as sedentary type activity during PE lessons using SOFIT. Muscular work of this nature might be likened to resistance work, which has been shown to elicit its own metabolic health benefits [32]. Our study may have been improved, and stronger inferences drawn on intervention effects on the intensity of PA had we been able to use accelerometers from the beginning of the study. However, given the random selection of schools with similar SES characteristics, large number of participants and the absence of group differences in pedometer derived PA, we have no reason to suspect group differences in MVPA and sedentary activity existed at baseline. Each of the measures of PA used in this study has limitations. Pedometers only record ambulatory activity and are not able to determine intensity and duration of activity. We used an accelerometer epoch length of 60s and it has been suggested that larger epoch lengths may under-report MVPA [33]. SOFIT observation times were pre-arranged to accommodate school timetables. Consequently, both control and intervention teachers had the opportunity to prepare classes in advance which could have influenced lesson delivery and content.

Another limitation was the difficulty of assessing the frequency of PE classes in the Control schools. Unlike Intervention schools, PE lessons were not routinely scheduled in the Control schools, being predominantly conducted on an ad hoc basis. In their annual questionnaires, Control school teachers frequently reported conducting 150 min per week of PE, the local government mandated level. We believe this to be inaccurate as this was not consistent with students’ written response to a question (as part of written questionnaire designed for the LOOK study but untested for validity and reliability) asking how many times they had PE during the last week). In the final year of the study, Control students reported taking part in two PE lessons per week on average, which would amount to 70 mins per week (based on SOFIT lesson length). As a result of this ambiguity, we were unable to compare objectively measured whole-day PA of the two groups on days of the week PE was conducted, although this limitation had no impact on the comparison of differences in daily PA when averaged over the 7 day monitor wear period*.* In order to throw some light on the amount of PE received in Control schools, our group has since carried out a study (Keegan RJ et al., unpublished observations) on the quality and quantity of PE in schools in this jurisdiction. This study utilised a “naturalistic” approach where teachers were unaware when PE observations would take place, thereby removing the possibility of unusual preparation or incidence of PE classes. This approach revealed that classroom teachers actually taught in the order of only 30 min of PE and sport per week, in contrast with the 150 min mandated by the curriculum. In effect the Control group in the present study was likely closer to a non-PE group than anticipated. It also suggests that classroom teachers of the Intervention students were unlikely to have provided more PE than that the two classes per week delivered by the visiting specialist teachers. It is therefore even more surprising that no intervention effects on PA were observed, supporting the premise that well-conducted school PE of two 45 min classes per week, appears to exert only a small influence on the overall PA of primary school children.

## Conclusion

This study showed that a specialist taught PE program, designed to achieve broad-based educational and health objectives, can provide higher levels of PA during PE lessons than the usual practice PE conducted by generalist class-room teachers; and this can make a small contribution to whole day PA. However, there was no evidence to suggest that this translated to an increase in daily habitual PA or an increase in the number of students meeting PA recommendations. A trend towards an intervention effect on sedentary behaviour in boys warrants further investigation into the role that PE can play in reducing sedentary behaviour. Our data suggest that for PE to be a significant contributor to improving PA in pre-adolescent children, daily classes are likely to be required, and that even this would need to be supplemented with a wider multicomponent PA strategy.

## Abbreviations

MVPA, moderate to vigorous physical activity; PA, physical activity; PE, physical education; SES, socio-economic status; SOFIT, system for observing fitness instruction time; TPA, total physical activity; VPA, vigorous physical activity

## Additional files

Additional file 1: Table S1. Characteristics of the Intervention. (DOCX 30 kb) Additional file 2: Table S2. Median values and Interquartile range for the proportion of lesson time and minutes per lesson spent on differing lesson content. (DOCX 31 kb)
