# Supplementary material for: Outcomes of a four-year specialist-taught physical education program on physical activity: a cluster randomized controlled trial, the LOOK study
Source: Int J Behav Nutr Phys Act. 2016 Jun 8;13:64. doi: 10.1186/s12966-016-0388-4 (PMC4897937; doi:10.1186/s12966-016-0388-4)
Supplement: Additional file 1: Table S1. — Characteristics of the Intervention. (DOCX 30 kb) [file 12966_2016_388_MOESM1_ESM.docx]

**Additional file 1: Table S1. Characteristics of the Intervention**

| **Name** | | Specialist-taught Physical Education Intervention |
| --- | --- | --- |
| **Provider** | | The Bluearth Foundation, a non-for-profit company. Bluearth website: [www.bluearth.org](http://www.bluearth.org) |
| **Program objectives** | | To deliver physical education lessons that increase student physical activity in an all-inclusive, enjoyable, challenging, yet not-threatening environment |
| **Theoretical Framework** | | Guided Discovery method of teaching [15] |
| **Child-related objectives** | | Personal, social and physical development through movement challenges. |
| **Delivery** | **Who** | Five university qualified PE teachers (3 male, 2 female) with additional training from Bluearth delivered the Intervention |
|  | **How** | Face to Face PE lessons, programmed into the school curriculum, taught by the specialist PE teachers |
|  | **Duration** | 2 x 45 minute PE lessons per week over 4 school years. On average students received 272 lessons over the 4 year intervention period (68 lessons per school year) |
| **PE lesson Elements** | **Fitness** | Coordination and Agility: Challenge based activities including crawling, climbing, running, hopping, stepping, skipping, rope-skipping and jumping  Dynamic Movements: Gymnastic-based activities to develop rhythm and balance. For example, double-leg squat jump to single-leg landing |
|  | **Skill Activities** | Group and individual practices to develop motor skills of hitting, kicking, throwing, juggling and catching with a variety of objects |
|  | **Games** | Designed to promote aerobic fitness, cooperation and teamwork, problem solving and healthy attitudes towards competition |
|  | **Core Movement** | Including yoga-like practices to develop muscular strength, flexibility, balance and postural control |
