# Supplementary material for: Outcomes of a four-year specialist-taught physical education program on physical activity: a cluster randomized controlled trial, the LOOK study
Source: Int J Behav Nutr Phys Act. 2016 Jun 8;13:64. doi: 10.1186/s12966-016-0388-4 (PMC4897937; doi:10.1186/s12966-016-0388-4)
Supplement: Additional file 2: Table S2. — Median values and Interquartile range for the proportion of lesson time and minutes per lesson spent on differing lesson content. (DOCX 31 kb) [file 12966_2016_388_MOESM2_ESM.docx]

**Additional file 2: Table S2. Median values and Interquartile range for the proportion of lesson time and minutes per lesson spent on differing lesson content**

| **SOFIT category (minutes per lesson)** | **Control (N=97)** | | |  | **Intervention (N=96)** | | |
| --- | --- | --- | --- | --- | --- | --- | --- |
|  | **Mdn** | **25%** | **75%** |  | **Mdn** | **25%** | **75%** |
| **LESSON CONTENT** |  |  |  |  |  |  |  |
| Management | 4.11 | 2.32 | 7.62 |  | 8.40 | 5.46 | 11.21 |
|  |  |  |  |  |  |  |  |
| General Knowledge | 1.01 | 0 | 4.18 |  | 8.38 | 2.18 | 12.17 |
| Physical Fitness Knowledge | 0.34 | 0 | 2.36 |  | 0 | 0 | 1.68 |
| Fitness Activity | 0.67 | 0 | 5.30 |  | 7.45 | 4.05 | 11.33 |
| Skill Practice | 3.04 | 0 | 11.65 |  | 4.10 | 0 | 10.83 |
| Game | 3.06 | 0 | 14.97 |  | 11.19 | 0 | 17.36 |
| **TEACHER BEHAVIOUR** |  |  |  |  |  |  |  |
|  |  |  |  |  |  |  |  |
| Promotes Fitness | 0.34 | 0 | 3.70 |  | 1.33 | 0.33 | 2.66 |
| Demonstrates Fitness | 2.00 | 0.16 | 4.07 |  | 11.66 | 7.36 | 17.75 |
| General Instruction | 10.33 | 6.38 | 17.74 |  | 17 | 10.12 | 24.00 |
| Manages | 5.92 | 3.38 | 10.83 |  | 8.77 | 5.73 | 12.12 |
| Observes | 1.67 | 0.50 | 4.42 |  | 1.01 | 0.34 | 3.19 |
| Other Task | 0.33 | 0 | 1.00 |  | 0 | 0 | 1.35 |
